# *GmPGL2*, Encoding a Pentatricopeptide Repeat Protein, Is Essential for Chloroplast RNA Editing and Biogenesis in Soybean

**DOI:** 10.3389/fpls.2021.690973

**Published:** 2021-09-09

**Authors:** Xingxing Feng, Suxin Yang, Yaohua Zhang, Cheng Zhiyuan, Kuanqiang Tang, Guang Li, Hui Yu, Jiantian Leng, Qingyu Wang

**Affiliations:** ^1^College of Food and Biological Engineering, Xuzhou University of Technology, Xuzhou, China; ^2^Key Laboratory of Soybean Molecular Design Breeding, Northeast Institute of Geography and Agroecology, The Innovative Academy of Seed Design, Chinese Academy of Sciences, Changchun, China; ^3^College of Plant Science, Jilin University, Changchun, China

**Keywords:** soybean, RNA editing, pentatricopeptide repeat protein, genetic mapping, chloroplast function

## Abstract

Chloroplast biogenesis and development are highly complex processes requiring interactions between plastids and nuclear genomic products. Pentatricopeptide repeat (PPR) proteins play an essential role in the development of chloroplasts; however, it remains unclear how RNA editing factors influence soybean development. In this study, a *Glycine max pale green leaf 2* mutant (*Gmpgl2*) was identified with decreased chlorophyll contents. Genetic mapping revealed that a single-nucleotide deletion at position 1949 bp in the *Glyma.05g132700* gene in the *Gmpgl2* mutant, resulting in a truncated GmPGL2 protein. The nuclear-encoded *GmPGL2* is a PLS-type PPR protein that localizes to the chloroplasts. The C-to-U editing efficiencies of *rps16*, *rps18*, *ndhB*, *ndhD*, *ndhE*, and *ndhF* were reduced in the *Gmpgl2* mutant. RNA electrophoresis mobility shift assay (REMSA) analysis further revealed that GmPGL2 binds to the immediate upstream sequences at RNA editing sites of *rps16* and *ndhB in vitro*, respectively. In addition, GmPGL2 was found to interact with GmMORF8, GmMORF9, and GmORRM6. These results suggest that GmPGL2 participates in C-to-U RNA editing *via* the formation of a complex RNA editosome in soybean chloroplasts.

## Introduction

The chloroplast is a vital photosynthetic organelle for plant growth and development. It is a semi-autonomous organelle with its own DNA genome. The chloroplast proteome contains approximately 3,000 proteins, and only approximately 160 proteins are encoded by the chloroplast genome while the remainder are imported (Martin et al., 2002). The proteins encoded by the chloroplast genome include components of the chloroplast ribosome and NADH dehydrogenase-like complex, which influence plastid protein synthesis and photosystem I cyclic electron transport, respectively (Laughlin et al., 2019). RNA editing plays an important role in the biogenesis and functioning of the mitochondria and chloroplasts. The conversion of cytidines (Cs) to uridines (Us), via a deamination reaction, representing the main RNA editing mechanism in plants (Stern et al., 2004). RNA editing converts hundreds of Cs to Us at specific positions in the plastid and mitochondrial transcripts; moreover, editing often creates start or stop codons (Stern et al., 2010; Small et al., 2020). Meanwhile, a lack of RNA editing may have severe consequences, such as impaired chloroplast biogenesis (Yu et al., 2009; Ma et al., 2017; Jiang et al., 2018; Lv et al., 2020) and embryo lethality (Li et al., 2014, 2019; Sun et al., 2015a).

Several proteins, including pentatricopeptide repeat (PPR) proteins (Barkan et al., 2012; Yin et al., 2013), multiple organellar RNA editing factors (MORF, also known as RIPs, RNA editing factor interacting proteins; Sun et al., 2013; Ma et al., 2017; Jiang et al., 2018), organelle RNA recognition motif (ORRM) proteins (Sun et al., 2013), organelle zinc-finger (OZ) proteins (Sun et al., 2015b), and protoporphyrinogen oxidase 1 (PPO1; Tillich et al., 2009), are involved in RNA editing. The PPR proteins are characterized by 31–36 amino acid (aa) tandem repeats that fold into a pair of anti-parallel alpha helices to facilitate specific binding to target RNA sequences (Fujii and Small, 2011; Barkan et al., 2012; Yin et al., 2013). Most PPR proteins are predicted to be localized to the chloroplast, mitochondrion, or both these organelles (Colcombet et al., 2013). The PPR proteins are further divided into P- and PLS class proteins based on their structure. The P-class proteins contain an array of canonical PPR (P) motifs with 35 aa that participate in RNA-processing activities; by regulating translation initiation, intron splicing, RNA maturation, and RNA stability (Kotera et al., 2005; Haili et al., 2016; Aryamanesh et al., 2017). The PLS-class is composed of not only canonical P motifs (35 aa) but also L (36 aa) and S (31 aa) variants (O’Toole et al., 2008). At their C-terminus, many PLS-class proteins extend to contain a plant-specific conserved E domain; half of the PLS proteins with this E domain are further extended to contain a DYW motif of 100 aa with cytidine deaminase (Schallenberg Ruedinger et al., 2013). The RNA editing reaction requires the C-terminal domains of the PLS-class proteins, as the E domain is essential for editing (Okuda et al., 2007, 2009; Chateigner Boutin et al., 2013; Hayes et al., 2013; Wagoner et al., 2015). Molecular and phylogenetic studies suggest that the terminal DYW domain of PLS-class proteins is also required for the editing activity (Boussardon et al., 2012). PLS-class proteins primarily participate in RNA editing in organelles. In addition, the PPR proteins are essential for the normal activities of the mitochondria and chloroplasts as the majority of the PPR protein mutants display varied physiological phenotypes, such as pigment deficiency (Pyo et al., 2013; Huang et al., 2018), photosynthetic defects (Cai et al., 2009; Johnson et al., 2010), seedling lethality (Sun et al., 2018; Li et al., 2019), and restricted growth (Sung et al., 2010; Hu et al., 2012; Xiao et al., 2018). Recent studies have shown that MORF2, MORF8, and MORF9 are localized to plastids and are required for chloroplast RNA editing (Yan et al., 2017; Huang et al., 2019; Zhang et al., 2019; Zhao et al., 2019); ORRM1 and ORRM6 are also localized to plastids and participate in chloroplast RNA editing (Searing et al., 2020).

Soybean is an important source of edible oil and proteins for human and animal nutrition. The demand for soybean is continuously increasing with the rapid increase in human consumption and industrial use of soybean products (Ainsworth et al., 2012). However, the current rate of increase in soybean yield is insufficient to meet the growing demand. An analysis of historical soybean germplasm revealed that breeders have increased soybean yield by improving the plant harvest index, canopy light interception, and seasonal conversion efficiency, as well as by effectively utilizing of solar energy for the production of plant biomass (Morrison et al., 1999; Koester et al., 2014). The recently released cultivars have a higher daily carbon gain, chlorophyll content, and sink capacity than older cultivars. However, the maximum photosynthetic capacity, mesophyll conductance, and nighttime respiration have remained unchanged (Koester et al., 2016). Recent advances in synthetic biology and molecular biology have enabled the development of technologies for redesigning photosynthesis, thereby meeting the global food and bioenergy demand (Zhu et al., 2020a). Therefore, it is crucial to understand the molecular basis of soybean chloroplast function for yield improvement, particularly with respect to certain gaps in knowledge, such as the role of RNA editing in the regulation of chloroplast genes. *Glyma.20G187000* encodes the chloroplast-localized protein ORRM1 that regulates chloroplast RNA editing and photosynthesis (Zhu et al., 2020b). The soybean genome encodes approximately 400 PPR proteins; however, the fundamental molecular functions of most of these proteins remain unknown (Su et al., 2019).

Herein, to identify novel factors involved in chloroplast development, a *Glycine max pale green leaf 2* (*Gmpgl2*) mutant was developed. Map-based cloning revealed that a chloroplast-localized GmPGL2 protein is mutated in *Glyma.05g132700*. GmPGL2, together with GmMORF8, GmMORF9, and GmORRM6, participates in chloroplast transcript editing. Our study provides evidence that the chloroplast-localized GmPGL2 proteins regulate the normal functioning of organelles, particularly the chloroplast.

## Materials and Methods

### Plant Materials and Chlorophyll Analysis

The *Gmpgl2* mutant was screened in June 2011 from the ^60^CO γ-radiation mutagenized Hedou12 (HD12) population as described previously (Cheng et al., 2016a). To purify the genetic background, the *Gmpgl2* mutant plants were backcrossed for four generations in the Chang-Chun experimental field of Northeast Institute of Geography and Agroecology, CAS.

We collected fresh leaves from 18-day-old plants and determined their chlorophyll content using a spectrophotometer as described previously (Feng et al., 2019). Chlorophyll fluorescence was measured using FluorPen (Czech). Minimal chlorophyll fluorescence (*F_0_*) was measured at 650nm following the storage of leaves in the dark for 30min. Maximal chlorophyll fluorescence (*Fm*) was measured during a 1-s pulse of saturated white light (2,500μmolm^−2^s^−1^). The maximum quantum yield of photosystem II electron transport was calculated using the following formula: *F_v_/F_m_ =(F_m_-F_0_)/F_m_*, where *F_v_* indicates the maximum variable fluorescence.

### Nuclear Acid Extraction and Analysis

Genomic DNA was extracted using the DNeasy Plant Mini Kit (Qiagen, Germany). The sequences of anchor markers used for initial mapping were published previously (Song et al., 2015). For fine mapping the *GmPGL2* locus, new primers of InDel markers were synthesized for polymerase chain reaction (PCR; Supplementary Table S1). The candidate genes were amplified by PCR, and the PCR products were sequenced by Sangon Biotech (Shanghai, China). The phylogenetic and syntenic analyses were carried out as described previously (Dai et al., 2018).

The total RNA was extracted from tissue samples using TRIzol reagent (Qiagen, Germany) according to the manufacturer’s instructions. RNA samples were reverse transcribed using primer Script I (TaKaRa, Japan). An 18-mer oligo (dT) primer for nuclear-encoded genes or random primers for plastid genes were used for first strand cDNA synthesis. Quantitative real-time PCR (qRT-PCR) was performed using the SYBR^®^ Premix Ex Taq^™^ Kit (TaKaRa, Japan) on an MX3005P Real-Time PCR System; the primers used are listed in Supplementary Table S1. The PCR program was as follows: 95°C for 15min, followed by 40cycles at 95°C for 10s, 58°C for 20s, and 72°C for 20s. *Actin11* was used as the reference gene (Jian et al., 2008). Three biological replicates were used for gene expression analysis.

### Plasmid Construction and Transformation

The CDS of *GmPGL2* was amplified from HD12 using KOD DNA polymerase (Toyobo, Japan); the PCR products were cloned into the TA cloning vector pMD18-T. The *GmPGL2* gene was cloned into the binary vector pCAMBIA3301 (CAMBIA, United States), using *Hind*III and *EcoR*I restriction endonuclease enzymes, and the pCAMBIA3301-*GmPGL2* plasmid construct was generated. This plasmid was introduced into *Agrobacterium tumefaciens* (strain EHA 105) for the transformation of cotyledonary explants of soybean (Gao et al., 2020).

### Bioinformatics Analysis

The homologs of GmPGL2 protein were characterized by BLAST tool in Phytozome^1^; the phylogenetic and microsyntynteny analysis were performed as described previously (Tang et al., 2020). The signal peptide of GmPGL2 protein was analyzed by TargetP2.0 program.^2^ The conserved motifs of GmPGL2 domain were predicted as described by Ian Small group (Cheng et al., 2016b; Gutmann et al., 2020). Target RNA sites prediction of GmPGL2 protein used the “PPR CODE PREDICTION WEB SERVER (Ver. 1.6.11)”^3^ (Yan et al., 2019). Some PPR codes are based on PPR code dataset from Kobayashi et al. (2019).

### Subcellular Localization and Microscopy

The full-length CDS and the 594-bp region (*GmPGL2*^1-198^) of the *GmPGL2* gene were fused to green fluorescent protein (GFP) at the C terminal, and then amplified and cloned into the modified 3301H vector at the *Xma*I and *Hind*III sites. We prepared two constructs, namely, *35:GmPGL2-GFP* and *35S:GmPGL2*^1-198^*-GFP*, which were introduced into *A. tumefaciens* (strain EHA105) and subsequently used to infiltrate *Nicotiana benthamiana* leaves as described previously (Waadt and Kudla, 2008). The GFP fluorescence signals were detected using a LSM510 laser scanning confocal microscope (Carl Zeiss, Germany). Transmission electron microscopy was performed according to a previously described method (Kwon and Cho, 2008).

### RNA Editing Analysis Through RNA-Sequencing

The total RNA was isolated from the leaves of 12- and 18-day-old HD12 and *Gmpgl2* seedlings. Thereafter, rRNAs were removed to retain mRNAs and non-coding RNAs (ncRNAs). The enriched mRNAs and ncRNAs were cut into short fragments in fragmentation buffer and reverse transcribed into cDNA with random primers. The second-strand cDNAs were synthesized using DNA polymerase I, RNase H, dNTPs, and buffer. Next, the cDNA fragments were purified using the QiaQuick PCR Extraction Kit and end-repaired. Poly(A) was added to the fragments, which were finally ligated to Illumina sequencing adapters. The ligation products were size selected by agarose gel electrophoresis, PCR amplified, and sequenced using Illumina HiSeq™ 4,000 by Gene Denovo Biotechnology Co. (China). The reads containing adapters and low-quality reads were removed, and RNA sequences were eliminated using the alignment tool Bowtie2 (Langmead and Salzberg, 2012). The remaining reads were considered for assembling contigs (cDNA sequences) for transcriptome analysis. The rRNA-mapped reads of each sample were then mapped to the reference genome using TopHAT2 version 2.0.3.12 (Kim et al., 2013). The transcripts of the chloroplast genes were identified by referring to the soybean chloroplast genome database.^4^ RNA editing of a gene was considered to occur if the fold-change in the mRNA variants with single-nucleotide polymorphisms (SNPs) at the editing sites was ≥2 for reference reads and ≥3 for variant reads, and the mutation frequency was between 0.1 and 0.9 (Bahn et al., 2012; Ramaswami et al., 2013). Three biological replicate samples were analyzed for each developmental stage.

### Recombinant Protein Expression and RNA Electrophoresis Mobility Shift Assay

The cDNA fragments of GmPGL2 were amplified with specific primers OL13194 and OL13195 (Supplementary Table S1) and cloned into the pCold vector (TaKaRa, Japan) to generate recombinant His-GmPGL2. The recombinant protein was purified across columns equipped with Ni2+ affinity resin (Ni-NTA Resin, GenScript; Supplementary Figure S1). RNA probes were synthesized and labeled with 6-FAM at the 3' end by GenScript (Nanjing, China). For REMSAs, the method was similar to a previously described protocol with little modification (Xiao et al., 2018). The recombinant protein was incubated with a labeled RNA probe in a reaction mixture including 2x binding buffer (100mM Na phosphate (pH 7.5), 10units RNasin, 0.1mgml^−1^ BSA, 10mM dithiothreitol, 2.5mgml^−1^ heparin, and 300mM NaCl). The mixture was incubated at 25°C for 30min followed by separation through 5% native polyacrylamide gel electrophoresis (PAGE) in 1xMOPS buffer (50mM MOPS, 50mM Tris–HCl, 1mM EDTA, pH7.3). After electrophoresis, the gels were imaged using a fluorescent biological image analysis system, Tanon 4600SF (Tianneng, China). Three concentrations (5, 10 and 50 pmol) of unlabeled probes were added to the reaction mixture as competitive probes.

### Yeast Two-Hybrid (Y2H) and Luciferase Complementation Imaging Assay

The Gal4-based Y2H assay was performed using the Matchmaker Gold Yeast Two-Hybrid System (TaKaRa, Japan) according to the manufacturer’s instructions. The cDNA of *GmPGL2* and mutated *GmPGL2* (*GmPGL2m*) were cloned into the GAL4-binding domain vector (pGBKT7-BD), and the cDNAs of *GmMORF1*, *GmMORF2*, *GmMORF8*, *GmMORF9*, *GmORRM1*, and *GmORRM6* were cloned into the GAL4 activation domain vector (pGADT7-AD). Combinations of constructs were co-transformed into the yeast strain Y2H Gold (TaKaRa, Japan). The co-transformants were cultured on SD/-Leu/-Trp and SD/-Ade/-His/-Leu/-Trp (QDO) media for 48h at 28°C to verify protein interactions.

The open reading frames of *GmPGL2* and *GmMORF8*, *GmMORF9*, and *GmORRM6* without stop codon were cloned into pCAMBIA1300-nLUC (NLUC) and pCAMBIA1300-cLUC (CLUC), respectively, yielding the GmPGL2-NLUC and MORF8-CLUC, and MORF9-CLUC and ORRM6-CLUC constructs, respectively. These constructs were introduced into *A. tumefaciens* (strain EHA105) and then used to infiltrate *N*. *benthamiana* leaves for the luciferase complementation imaging (LCI) assay as described previously (Wang et al., 2020). After incubation for 48h under a 16-h light/8-h dark cycle, the leaves were injected with D-luciferin at the final concentration of 1mM. Luciferase signals were imaged using the Tanon 4600SF system (Tianneng, China). GmAPC13a and GmILPA1 (Gao et al., 2017) were used as the positive controls for Y2H and LCI analysis.

### Statistical Analyses

All samples had at least three biological replicates. The statistical analyses were performed using R software (version 3.6.2) as described previously (Tang et al., 2020). Asterisks indicate significant differences as determined by *p* values (^*^p<0.05; ^*^p<0.01; and ^**^p<0.001).

## Results

### Isolation and Phenotypic Characterization of the *Gmpgl2* Mutant

**Figure 1:**
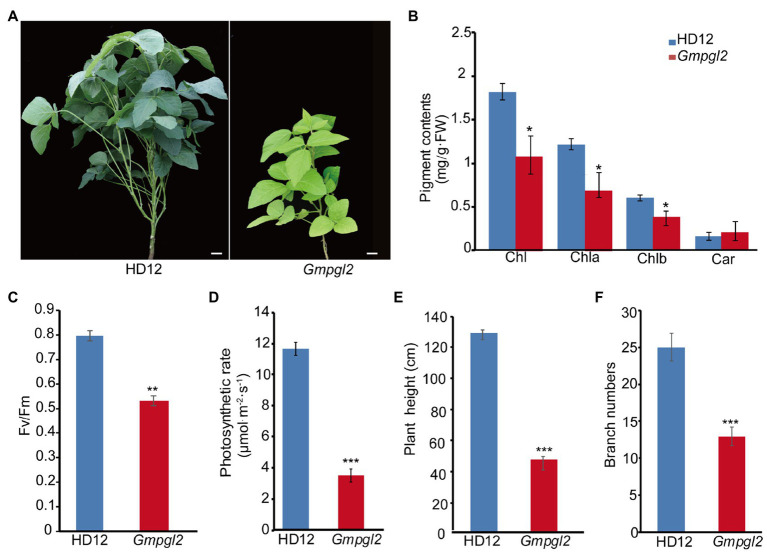
Phenotypes of Hedou12 (HD12; wild type) and *Gmpgl2* (mutant) plants. **(A)** Leaves of HD12 and *Gmpgl2*. Bar=5cm. **(B)** Pigment content in 18-day-old HD12 and *Gmpgl2* seedlings. *Chla*, chlorophyll a; *Chlb*, chlorophyll b; and *Car*, carotenoid. **(C)** Photosystem II maximum quantum yield (*F_v_/F_m_*) photosynthetic rate of 18-day-old HD12 and *Gmpgl2* seedlings. **(D)** Photosynthetic rate of 18-day-old HD12 and *Gmpgl2* seedlings. Values are mean±SE of three biological replicates. Asterisks denote significant differences from HD12, as determined using Student’s *t*-test. **(E)** Height of HD12 and *Gmpgl2* mutant plants. **(F)** Number of branches in HD12 and *Gmpgl2* mutant plants.

To elucidate the mechanism of chloroplast development in soybean, we isolated the *Gmpgl2* mutant by screening nearly 10,000 ^60^Co-γ radiation-induced M_2_ mutants for pale green leaf from a mutagenesis population of HD12 (Cheng et al., 2016a). The pale green leaves of the *Gmpgl2* mutant were clearly identified upon the emergence of the first true leaf and throughout the developmental process (Figure 1A). The components of total chlorophyll (Chl), chlorophyll a (Chla), and chlorophyll b (Chlb) in the leaves of the *Gmpgl2* mutants decreased by 40.4, 42.7, and 35.7% of the respective values in the wild-type leaves. The carotenoid (Car) content in the leaves of the mutant plants was 80% that in the leaves of wild-type plants (Figure 1B). Furthermore, the ratio of Chl/Car in the *Gmpgl2* mutant was lower than in the wild type, which might be due to a substantial decrease in chlorophyll synthesis compared to carotenoid synthesis.

The photosystem II maximum quantum yield of *Gmpgl2* was only 67.1% of that of HD12, and the photosynthetic rate of *Gmpgl2* was 3.41±0.39μmol^−1^m^2^·s^−1^, representing 70.6% of that of HD12 (Figures 1C,D). The *Gmpgl2* plants were shorter with fewer branches than HD12 plants (Figures 1E,F). Together, these results showed that the mutation in *GmPGL2* leads to defects in chloroplast biogenesis.

### Genetic Mapping Reveals That *GmPGL2* Encodes a PPR Protein

**Figure 2:**
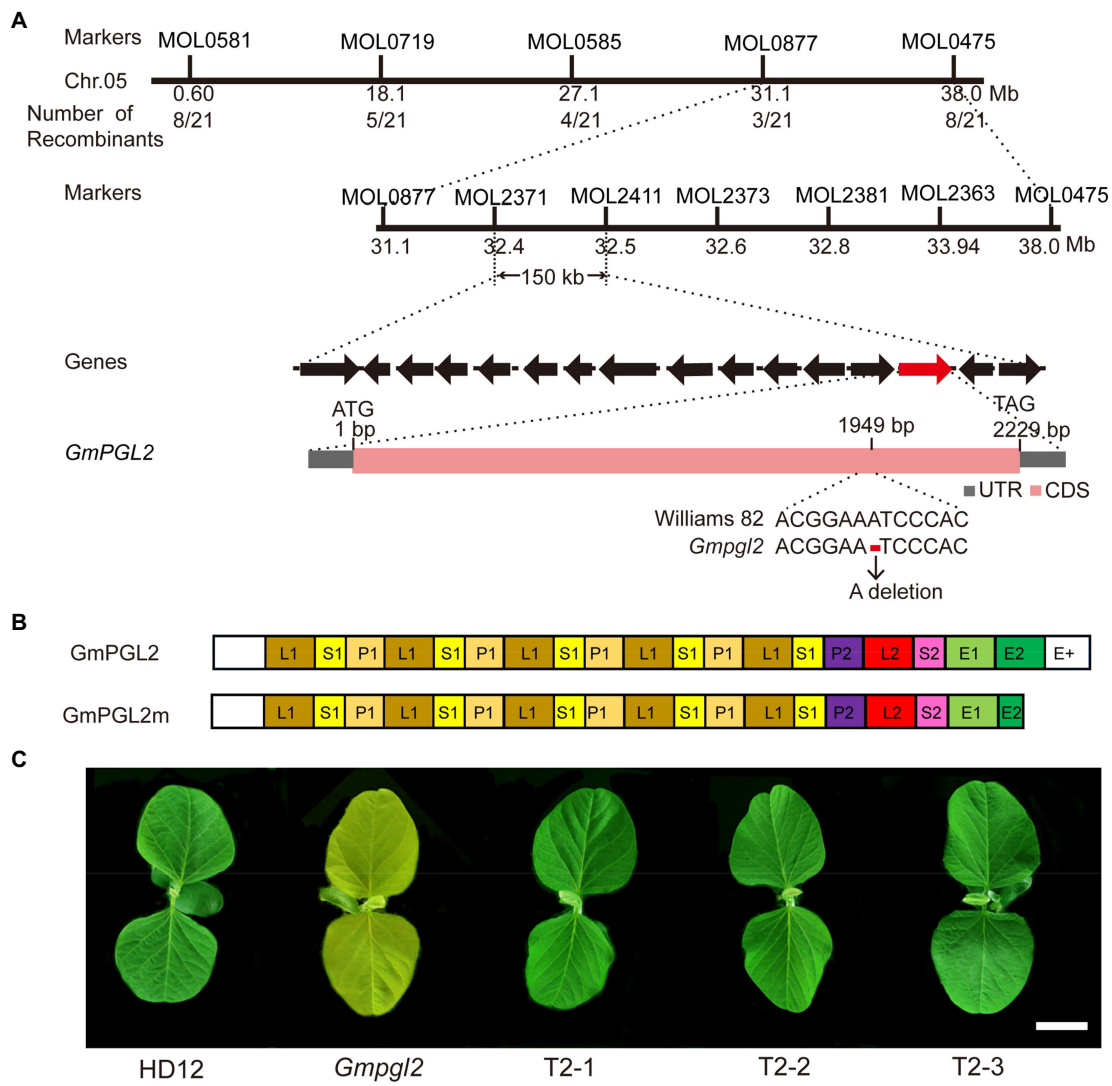
Map-based cloning of the *GmPGL2* gene. **(A)** Map-based cloning of the *GmPGL2* allele. (i) The *GmPGL2* mutation was narrowed to a 150-kb region between the InDel markers MOL2371 and MOL2411 on chromosome 5. (ii) The black arrows represent the 15 putative genes in this 150-kb genomic region; the candidate gene *GmPGL2* (*Glyma.05g132700*) is indicated with the red arrow. (iii) Schematic diagram of the *Glyma.05g132700* gene. ATG and TAG, the start and stop codons, respectively, are shown. A deletion point mutation at the 1949bp nucleotide position is shown. **(B)** Schematic diagram of the GmPGL2 protein. The *GmPGL2* gene encodes a PPR protein of the PLS-type carrying a E+ domain. The GmPGL2 protein has 17 PPR motifs; P, L, and S represent the PPR motifs of various repeat lengths; E+ is a domain required for RNA editing. **(C)** Complementation of the *Gmpgl2* mutant. Phenotypes of HD12, *GmPGL2*, and a 35S:*GmPGL2* complemented *Gmpgl2* plant. Scale bars=3cm.

In an attempt to identify the *GmPGL2* gene, the *Gmpgl2* mutant was crossed with the cultivar Williams 82 to generate a segregation population for mapping. The F_1_ plants were normal, and the F_2_ plants segregated in the ration of 3:1 (green:pale green=210:60; *χ*^2^=0.53; *p*>0.05), indicating that the *Gmpgl2* phenotype was controlled by a single recessive nuclear gene. Using 165 insertion/deletion (InDel) markers developed earlier (Song et al., 2015), the *GmPGL2* locus was initially mapped to a 6.9-Mb region between the MOL0877 and MOL0475 markers of chromosome 5 (Figure 2A). Thereafter, the *GmPGL2* gene was further mapped to a 150-kb region between markers MOL2371 and MOL2411 using 702 F_3_ individuals with the pale green phenotype. Fifteen genes in this region were predicted according to the annotation in the reference genome of Williams 82 (*Glycine max Wm82.a2.v1, see footnote 1*). Analyses of the coding sequences of these 15 genes in HD12 and *Gmpgl2* revealed a single adenine (A) deletion at 1949bp of the *Glyma.05g132700* gene resulting in a frame-shift mutation (Figure 2A; Supplementary Table S2).

To further confirm that the mutation in the *Glyma.05g132700* gene was responsible for the pale green leaf phenotype of *Gmpgl2*, the coding sequence (CDS) of *Glyma.05G132700*, driven by the cauliflower mosaic virus 35S promoter, was transformed into the *Gmpgl2* mutant *via A. tumefaciens*. Three independent transgenic lines carrying the *35S:GmPGL2* expression cassette were obtained in the *Gmpgl2* background. All transgenic lines completely rescued the *Gmpgl2* phenotype (Figure 2C), confirming *Glyma.05g132700* as *GmPGL2*.

### *GmPGL2* Encodes a PPR Protein Localized to the Chloroplast

Analyses of the deduced GmPGL2 amino acid sequence indicated that the GmPGL2 protein contains 17 PPR motifs, an E1/E2 domain, and a E+ domain, and is thus classified as a PPR-E+ subclass protein, similar to that in other PPR proteins reported previously (Rivals et al., 2006). A single-nucleotide deletion at 1949bp of its CDS (Figure 2A) results in a frame-shift mutation and generates a truncated protein that lacks a portion of the of E2 and E+ motif (Figure 2B).

**Figure 3:**
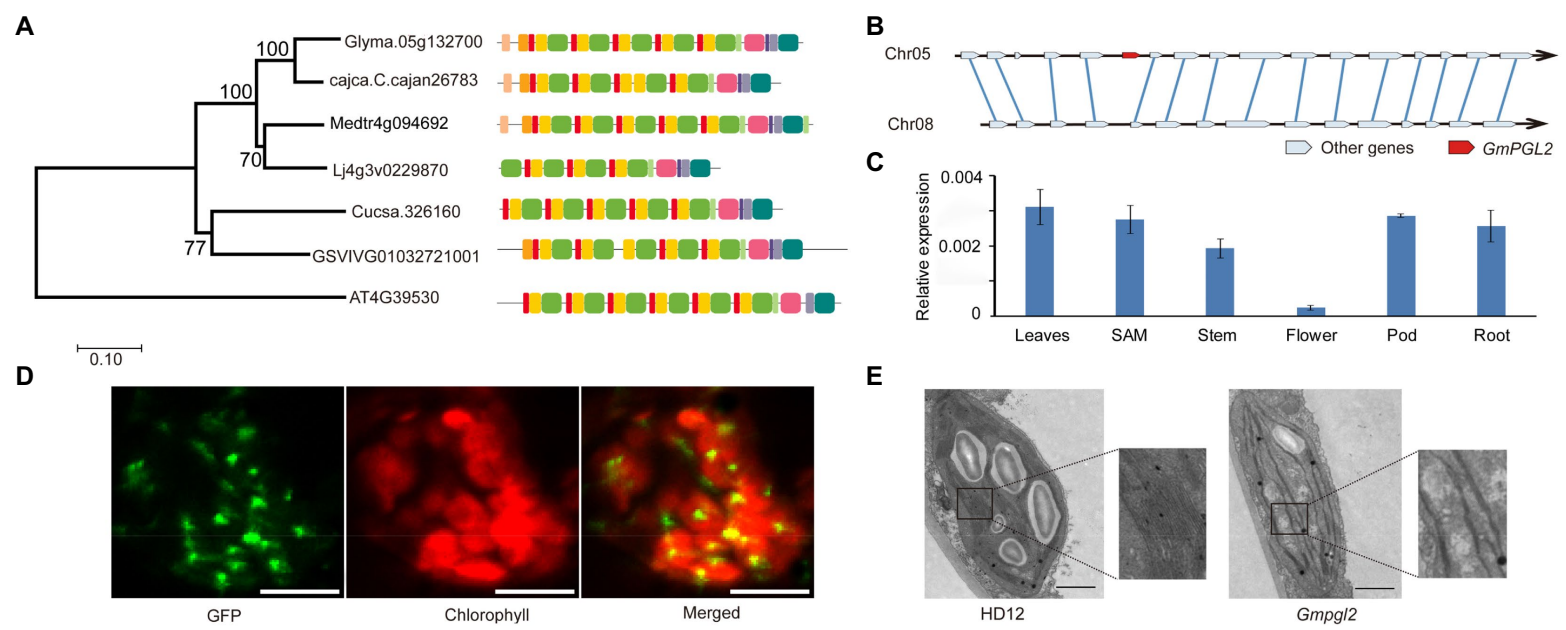
*GmPGL2* is a single copy gene. **(A)** Phylogenetic tree of GmPGL2 and homologous GmPGL2 proteins from *Arabidopsis thaliana*, *Medicago truncatula*, *Lotus corniculatus*, *Cajanus cajan*, *Cucumis sativus*, and *Vitis vinifera*, constructed using MEGA 5 with the neighbor-joining method. **(B)** Synteny plot of the homologous *GmPGL2* regions. Solid blue lines show the pairs of homologous genes of the two homologous chromosomes. **(C)** Expression of *GmPGL2* in different tissues. **(D)** Subcellular localization of GmPGL2. GFP signals of the 3301H-*GmPGL2*^1-198^-GFP fusion protein were localized to the chloroplast of epidermal cells of *N. benthamiana* leaves. Green fluorescence signals, chlorophyll auto-fluorescence signals, and a merged image are shown. Bar=10μm. **(E)** Transmission electron micrographs of the chloroplasts of 18-day-old HD12 and *Gmpgl2* seedlings. Bar=2μm.

The GmPGL2 protein sequence was used to identify its homologs in *Arabidopsis thaliana*, *Medicago truncatula*, *Lotus corniculatus*, *Cajanus cajan*, *Cucumis sativus*, and *Vitis vinifera* to construct a phylogenetic tree. GmPGL2 showed 74.3, 75.3, and 80.1% identity with homologs *cajca.C.cajan26783*, *Medtr4g094692*, and *Lj4g3v0229880* of *C. cajan*, *M. truncatula*, and *L. corniculatus*, respectively (Figure 3A). Following whole genome duplication, it is expected that soybean would carry two homologous *GmPGL2* genes. However, only one *GmPGL2* copy was observed in the phylogenetic analysis. To confirm this finding, we performed synteny analysis of the 143,208-bp region around *GmPGL2*. The syntenic analysis results revealed that 32,522,450–32,670,694bp of chromosome 5 and 6,566,793–6,686,020bp of chromosome 8 are more likely duplicated blocks in the soybean genome. As expected, no homolog of *GmPGL2* was found on chromosome 8 (Figure 3B). These results suggested that *GmPGL2* is a single copy gene in the soybean genome. To further understand the function of *GmPGL2*, the expression levels of *GmPGL2* in different tissues were examined using qRT-PCR. *GmPGL2* was expressed in all tested tissues and at higher levels in leaf than in stem, flower, pod, and root (Figure 3C), suggesting that *GmPGL2* may have a vital role in leaves.

TargetP^5^ prediction analysis showed a chloroplast-targeting signal at the N terminus (1–198 aa) of the GmPGL2 protein. To localize GmPGL2, the full-length GmPGL2 (without a stop codon) was fused with the GFP and transformed into *N. benthamiana*; however, no fluorescence signal was detected in the leaf cells of *N. benthamiana*. We suspected that full-length GmPGL2 with GFP may have been too large to be efficiently expressed, or over-expression of the full-length protein may be detrimental to the cells. Subsequently, a shortened 198-aa N-terminal sequence was fused with GFP (35S,GmPGL2^1-198^-GFP) to detect its localization, and the fluorescent signals were detected as punctuated dots localized to chloroplasts (Figure 3D). No GFP signal was detected in other compartments of the cell, suggesting that the GmPGL2 protein is localized to the chloroplast. The phenomenon of full-length PPR protein fused to GFP without a fluorescence signal has been observed previously (Wang et al., 2019).

Next, we observed the chloroplast ultrastructure of HD12 and *Gmpgl2* leaves. In the mesophyll cells of HD12 leaves, chloroplasts showed typical structures with continuous stacking of the grana (Figure 3E). In contrast, the ultrastructural analysis of chloroplasts in the leaves of the mutant showed less stacking of the grana than HD12 (Figure 3E).

### *GmPGL2* Is Required for the C-to-U Editing During Leaf Development

To understand the role of RNA editing in the development of soybean leaves, we analyzed the variations in chloroplast RNA sequences obtained from high throughput RNA-Seq data between HD12 and *Gmpgl2* at each of the following two developmental stages: (i) Stage 1 (S1): first trifoliate leaf of 12-day-old seedlings and (ii) Stage 2 (S2): first trifoliate leaf of 18-day-old seedlings.

Based on SNPs of the observed chloroplast RNA sequences and referenced sequences, 43 candidate RNA editing target sites were identified in the two stages in HD12 (Table 1). Thirty-eight of the 43 RNA editing sites were found to be located in the coding regions of 17 genes; whereas the other were located in the downstream, intergenic, and intron regions of *ndhK*-12704, *ndhJ*-14209, *rps12*-106113, *rps12*-138416, and *rps16*-55714, respectively. The 36 editing sites result in amino acid changes, except for *ndhC*-10779 and *petB*-74300. These editing sites result in six types of amino acid changes, namely, serine to leucine, proline to leucine, serine to phenylalanine, threonine to methionine, histidine to tyrosine, and threonine to leucine. We then compared the editing efficiency of HD12 and *Gmpgl2* during these two developmental stages.

**Table 1:** RNA editing efficiency between the HD12 and *Gmpgl2* seedlings.

Gene name	Editing sites	HD12-S1	HD12-S2	*Gmpgl2*-S1	*Gmpgl2*-S2	Amino acid change
ndhC	10779	29.58%	28.57%	28.98%	28.73%	Unchanged
ndhC	11062	95.76%	97.45%	94.38%	91.28%	Ser-Leu
ndhK	12704	2.89%	11.38%	0.41%	4.91%	Downstream
ndhJ	14209	2.88%	17.12%	2.62%	10.17%	Intergenic
rps14	23651	87.54%	75.43%	77.29%	68.19%	Ser-Leu
rpoB	34587	63.16%	52.80%	66.03%	52.01%	Ser-Phe
rpoB	34800	60.97%	70.40%	71.28%	63.21%	Ser-Leu
rpoB	34815	59.00%	75.71%	63.50%	70.22%	Ser-Leu
rpoB	36249	89.79%	86.59%	80.93%	80.49%	Ser-Phe
rpoC1	37529	75.08%	75.36%	75.79%	67.91%	Ser-Leu
rpoC1	38778	74.02%	62.27%	60.08%	47.24%	Ser-Leu
rpoC2	43303	3.32%	15.51%	3.11%	2.56%	Ser-Leu
rps2	45133	94.23%	95.49%	87.95%	85.52%	Thr-Ile
rps2	45247	87.55%	93.77%	81.33%	80.77%	Ser-Leu
atpF	48596	86.50%	90.61%	72.21%	74.35%	Pro-Leu
rps16	55714	81.84%	89.00%	90.59%	76.07%	intron
rps16	56313	79.02%	77.48%	0.00%	0.00%	Ser-Leu
accD	57518	87.08%	90.13%	85.64%	74.74%	Ser-Leu
psaI	58512	89.09%	91.48%	85.18%	83.38%	His-Tyr
rps18	66641	84.39%	72.97%	57.89%	50.63%	Ser-Leu
petB	74300	26.13%	26.77%	22.00%	24.78%	Unchanged
petB	74899	93.59%	96.18%	93.81%	88.49%	Ser-Leu
rps12	106113	36.84%	0%	0.00%	0%	Intergenic
ndhA	115982	79.04%	89.52%	77.35%	84.16%	Ser-Leu
ndhA	117982	62.86%	89.87%	63.66%	85.68%	Ser-Phe
ndhE	119873	64.74%	80.82%	42.93%	52.57%	Pro-Leu
ndhD	120618	41.35%	83.96%	18.75%	55.69%	Thr-Met
ndhD	120999	73.33%	84.51%	57.33%	71.82%	Thr-Ile
ndhD	121290	65.86%	80.65%	69.50%	82.43%	Ser-Leu
ndhD	121494	64.94%	81.79%	55.67%	78.83%	Ser-Leu
ndhD	121914	44.95%	46.69%	28.91%	37.30%	Ser-Leu
ndhF	124681	44.09%	87.06%	35.59%	55.98%	Ser-Leu
rps12	138416	80.00%	71.91%	51.23%	49.59%	Intron
ndhB	139627	92.33%	80.00%	68.57%	78.67%	Ser-Leu
ndhB	140020	83.63%	96.65%	74.06%	90.27%	Thr-Met
ndhB	140064	64.08%	91.35%	50.08%	81.80%	His-Tyr
ndhB	140215	73.86%	92.19%	47.35%	71.96%	Pro-Leu
ndhB	140224	76.93%	89.32%	61.82%	72.92%	Ser-Phe
ndhB	140999	78.49%	91.12%	61.73%	75.21%	Ser-Leu
ndhB	141005	82.14%	90.21%	60.49%	66.95%	Ser-Leu
ndhB	141281	77.03%	87.68%	53.58%	59.70%	Ser-Leu
ndhB	141424	61.89%	84.78%	33.91%	51.22%	His-Tyr
ndhB	141650	69.47%	91.50%	49.78%	79.97%	Pro-Leu

In S1, the editing efficiency of 20 editing sites decreased by more than 10% in *Gmpgl2* compared with that in HD12; these sites were *atpF*-48596; *ndhB*-139627, -141281, -141424, -141005, -140215, -140224, -140999, -140064, and -141650; *ndhD*-120618, -121914, and -120999; *ndhE*-119873; *rpoC1*-38778; and *rps14*-23651, *rps16*-56313, and *rps18*-66641. Two of which (*rps12-106113* and *rps12-138416*) were located in the intron region, whereas the remaining editing sites caused CDS alterations in 13 genes (Table 1).

In S2, five of the above editing sites (*ndhB*-140064 and -139627, *ndhD*-121914, *rps14*-23651, and *rps12*-106113) restored to less than 10% of the RNA editing efficiency between the HD12 and mutant seedlings, whereas five new editing sites (*ndhF*-124681, *accD*-57518, *rps2*-45247, *rpoC2*-43303, and *rps16*-55714) reduced more than 10% of the editing efficiency of the HD12 seedlings. These newly detected five editing sites led to four amino acids changes, as *rps16*-55714 was located in the intron region. Another 20 editing sites of 12 genes altered the editing efficiency of the *Gmpgl2* seedlings during development stage 2 (Table 1).

**Figure 4:**
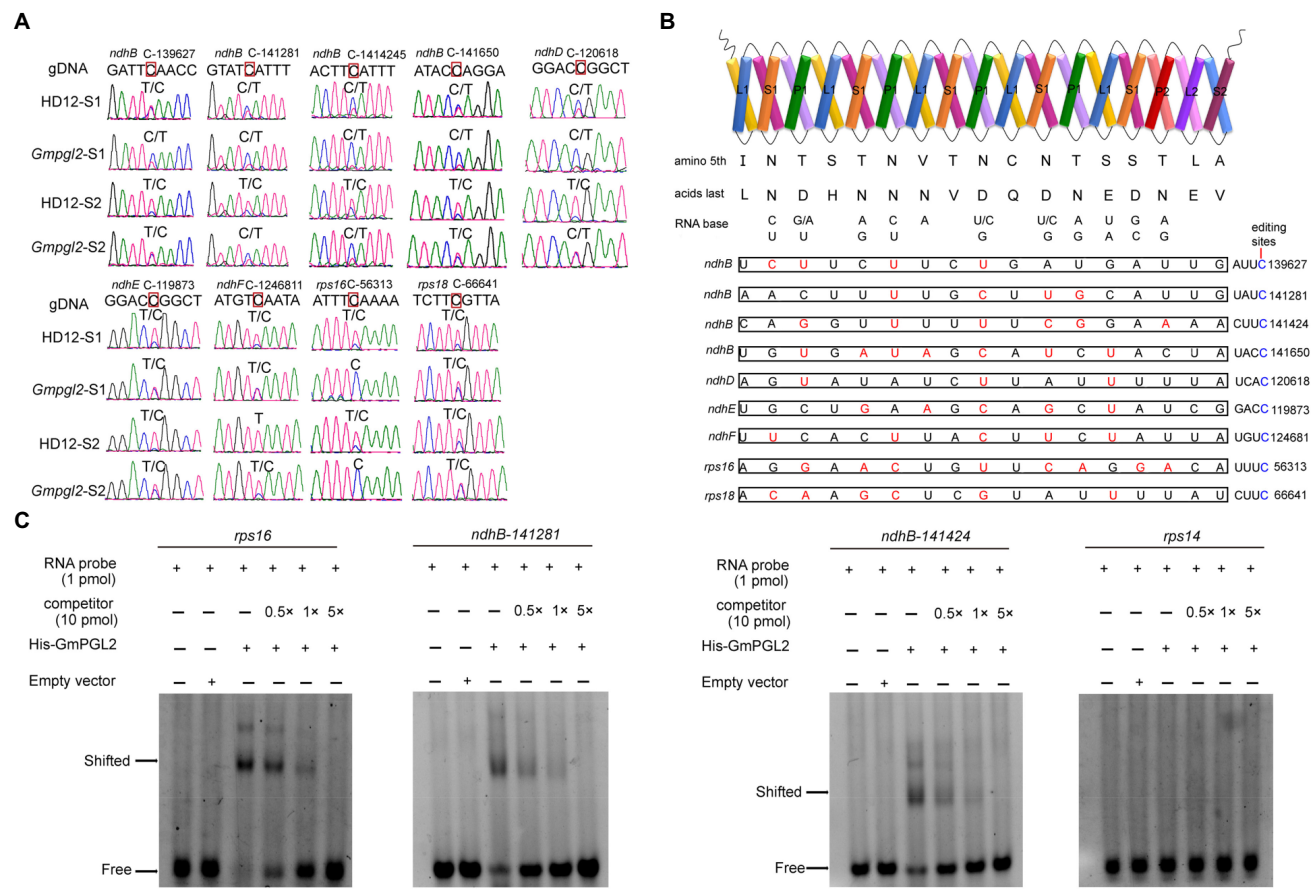
Analysis of GmPGL2 targeted genes and their interactions. **(A)** RNA editing sites validated by sequencing of the RT-PCR products. Green, black, red, and blue peaks represent A, G, T, and C, respectively. S1, leaves of 12-day-old seedlings; S2, leaves of 18-day-old seedlings. The names of the RNA editing sites are shown on top of the chromatograms. The edited bases are indicated by red square. **(B)** Schematic representation of GmPGL2 bound to its target sites. The combinations of positions 5 and last amino acid in each PPR motif of GmPGL2 were aligned to the nucleotide upstream of the editing site. Permissible matched nucleotides are indicated in red, and the edited sites C are indicated in blue. **(C)** REMSA of GmPGL2 protein with its target probes, *rps16*, and *ndhB*-141281, 141424. *rps14* probe was served as a negative control.

To validate the editing sites detected in chloroplast RNA sequences, we sequenced the cDNA of the above 36 amino acid-changed chloroplast editing sites (Figure 4A and Supplementary Figure S2). Nine putative editing sites were verified to differ between the HD12 and *Gmpgl2* seedlings in the two developmental stages: *ndhB*-139627, -141281, -141424, and -141650; *ndhD*-120618; *ndhE*-119873; *ndhF*-124681; *rps16*-56313; and *rps18*-66641. The editing site of *rps16*-56313 was altered most drastically. In HD12, the transcripts were edited in both developmental stages, whereas no editing was detected in *Gmpgl2*. The editing sites of *ndhB*-139627, -141281, -141424, -141650; *ndhD*-120618; *ndhE*-119873; *ndhF*-124681; and *rps18*-66641 were also reduced in the two developmental stages in the *Gmpgl2* mutant compared with those in HD12 (Figure 4A). The C-to-U editing caused serine to leucine substitutions in the *ndhB*-139627, *ndhB*-141281, *ndhF*-124681, *rps16*-56313, and *rps18*-66641 transcripts; a change from histidine to tyrosine in the *ndhB*-141424 transcripts; a change from proline to leucine in *ndhB*-141650 and *ndhE*-119873 transcripts, and a change from threonine to methionine in the *ndhD*-120618 transcripts.

Above results implied that *GmPGL2* participated in nine editing sites (*ndhB*-139627, -141281, -141424, -141650, *ndhD*-120618, *ndhE*-119873, *ndhF*-124681, *rps16*-56313, and *rps18*-66641) during young leaf development of soybean, and the *rps16*-56313 was completely abolished in the *Gmpgl2* mutant.

### GmPGL2 Protein Can Directly Bind to Its Targets *in vitro*

To evaluate the PPR-RNA recognition model, we analyzed the associations between GmPGL2 and chloroplast genes (Figure 4B). We found that the match indices between GmPGL2 and RNA editing efficiency of above nine sites, *ndhB*-139627, -141281, -141424, -141650, *ndhD*-120618, *ndhE*-119873, *ndhF*-124681, *rps16*-56313, and *rps18*-66641, were 4/17, 4/17, 6/17, 7/17, 3/17, 5/17, 5/17, 8/17, and 6/17, respectively (Figure 4B).

To further confirm that GmPGL2 actually binds to above sites, *ndhB-141281*, *ndhB*-141424, and *rps16*-56313 were selected to carry out the REMSA with the FAM-labeled RNA probes. The retarded bands appeared when His-GmPGL2 protein was incubated with these three labeled probes, however only free RNA probe bands were detected with the *rps14* probe as the negative control. The binding capacity to the labeled probes gradually decreased following the increased competitor concentration (Figure 4C), indicating that GmPGL2 binds to *ndhB*-141281, *ndhB*-141424, and *rps16*-56313 directly *in vitro*.

### *GmPGL2* Influences the Expression of Nuclear- and Plastid-Encoded Genes and Regulates Chloroplast Development

**Figure 5:**
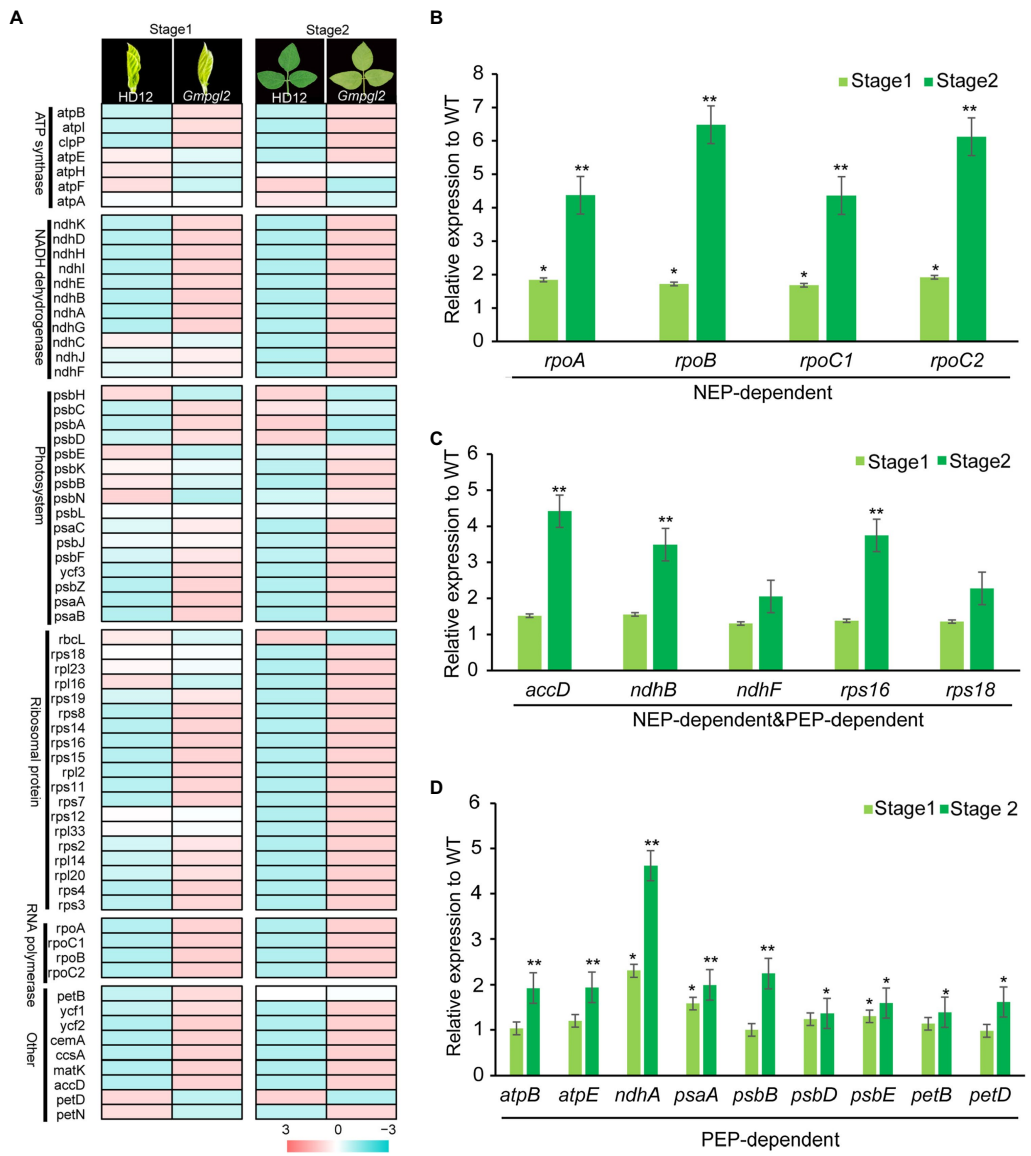
**(A)** Expression of chloroplast genes in the leaves of 12-day-old (Stage1) and 18-day-old (Stgae2) HD12 and *Gmpgl2* seedlings. **(B-D)** Comparison expression levels of chloroplast genes transcribed by NEP **(B)**, both NEP and PEP **(C)**, and PEP **(D)** between HD12 and *Gmpgl2* seedlings. The values are the mean ratio±standard deviation with three biological repeats. The asterisks indicate significant differences between WT and *Gmpgl2* (Student’s test; ^*^p<0.05; ^*^p<0.01).

In each of the two developmental stages, 66 chloroplast genes in HD12 and *Gmpgl2* seedlings were detected by RNA-Seq (Figure 5A). In the S1 developmental stage, a pairwise comparison of genes between HD12 and *Gmpgl2* seedlings revealed 11 differentially expressed genes (DEGs), namely, *rps15*, *rps16*, *rpl2*, *rpoA*, *rpoC2*, *ndhA*, *ndhG*, *ycf2*, *matK*, *psbZ*, and *cemA*. Meanwhile, in the S2 developmental stage, 30 DEGs were detected, namely *accD*, *matK*, *ndhA*, *ndhB*, *ndhD*, *ndhG*, *ndhH*, *ndhI*, *psaC*, *rpl2*, *rpl14*, *rpl16*, *rpl20*, *rpl23*, *rps2*, *rps3*, *rps4*, *rps7*, *rps8*, *rps11*, *rps12*, *rps15*, *rps16*, *rps19*, *rpoA*, *rpoB*, *rpoC1*, *rpoC2*, *ycf1*, and *ycf2* (Figure 5A). Most of DEGs encoded different components of the chloroplast-specific ribosomal proteins, suggesting that the mutation of *GmPGL2* influenced the expression of a large number of chloroplast genes.

The expression of chloroplast-encoded genes is strongly associated with chloroplast development, and it is coordinately transcribed by nuclear-encoded RNA polymerase (NEP) and plastid-encoded RNA polymerase (PEP; Huang et al., 2018; Xiao et al., 2018). To investigate the role of *GmPGL2* in chloroplast development, the expression levels of PEP- and NEP-dependent genes were compared between *Gmpgl2* and HD12 seedlings. The transcription levels of the most selected genes in *Gmpgl2* were upregulated in the young leaf developmental stage 2 compared with HD12 (Figures 5B–D). The results were consistent with those of RNA-Seq, suggesting that *GmPGL2* most likely influences the expression of nuclear- and plastid-encoded genes during chloroplast development.

### GmPGL2 Interacts With MORF8, MORF9, and ORRM6

**Figure 6:**
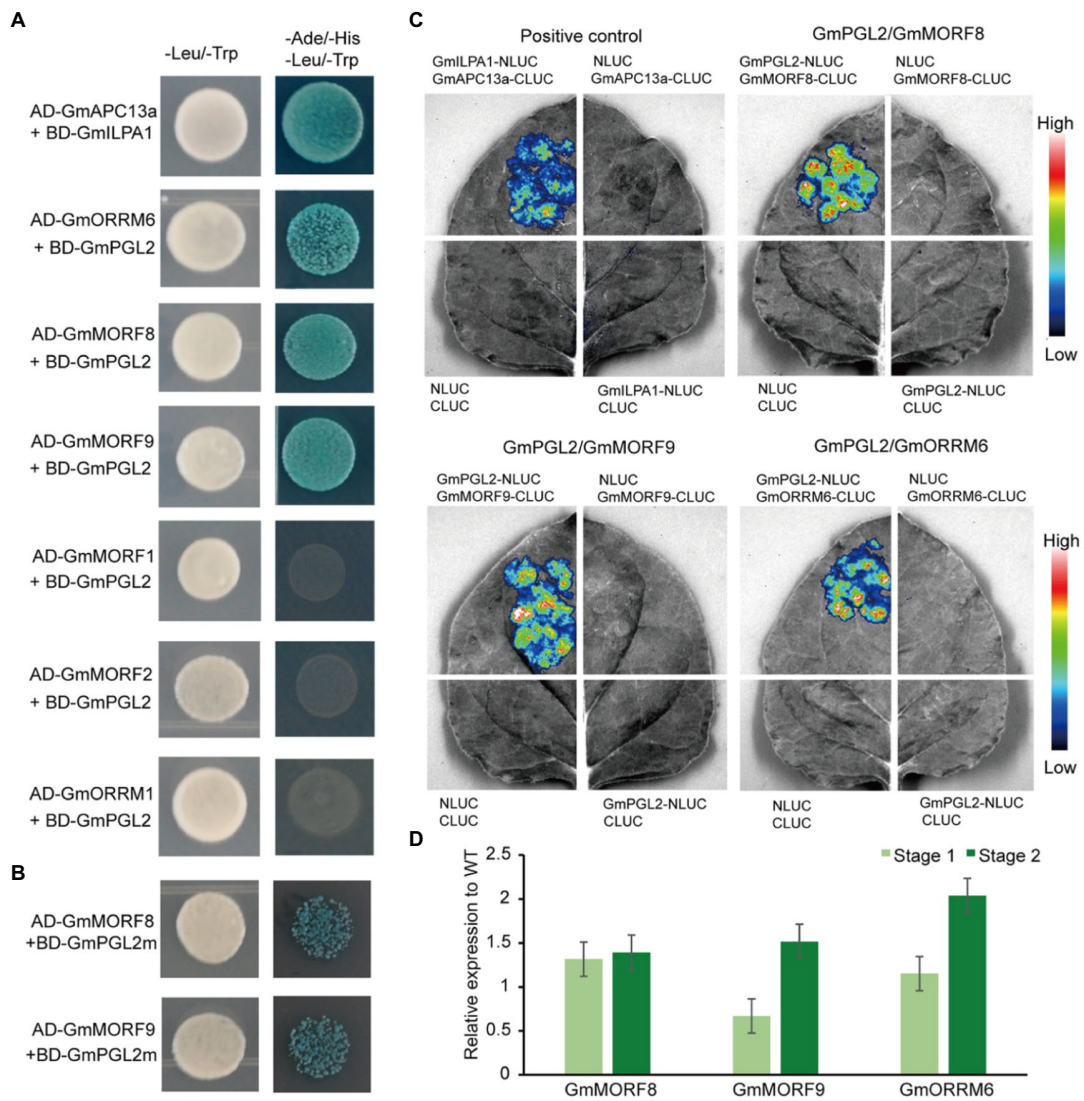
GmPGL2 interacts with GmMORFs and GmORRMs. GmAPC13a and GmILPA1 represent the positive control. **(A)** Yeast two-hybrid assay between GmPGL2 and some GmMORFs and GmORRMs. **(B)** Yeast two-hybrid assay between mutated GmPGL2 (GmPGL2m) and GmMORF8, GmMORF9, respectively. **(C)** Luciferase complementation imaging was performed to confirm the interactions between GmPGL2 and GmMORF8, GmMORF9, and GmORRM6 in *N. benthamiana*, respectively. The fluorescent signal intensity represents the strength of interaction. **(D)** The expression levels of GmMORF8, GmMORF9, and GmORRM6 during two developmental stages of young leaves in wild type and *Gmpgl2*. The values are the mean ratio±standard deviation with three biological repeats. None significant difference was determined by Student’s *t*-test between wild type and mutant.

To investigate GmPGL2 interaction proteins, the candidates of MORF and ORRM proteins were identified by searching for homologs from the relevant database (see footnote 1). Four MORF proteins and two ORRM proteins were selected to detect *in vitro* protein interaction through yeast two-hybrid assay, and they are GmMORF1 (*Glyma.08G188700*), GmMORF2 (*Glyma.04G042700*), GmMORF8 (*Glyma.13G271400*), GmMORF9 (*Glyma.15G064300*), GmORRM1 (*Glyma.20G187000*), and GmORRM6 (*Glyma.16G217600*; Figure 6A). GmMORF8/GmPGL2, GmMORF9/GmPGL2, and GmORRM6/GmPGL2 co-transformants were able to grow in the quadrupole drop-out (QDO) medium, whereas GmMORF1/GmPGL2, GmMORF2/GmPGL2, and GmORRM1/GmPGL2 co-transformants could not. We also found that the interactions of mutated GmPGL2 (GmPGL2m) protein with GmMORF8 and GmMORF9 were decreased (Figure 6B). These results suggested that the mature form of GmPGL2 interacts with GmMORF8, GmMORF9, and GmORRM6, but not with GmMORF1, GmMORF2, and GmORRM1.

Luciferase complementation imaging was performed to further investigate these interactions. High luciferase activity was detected after the co-expression of GmMORF8, GmMORF9, and GmORRM6 fused to C-terminal luciferase (CLUC) and GmPGL2 fused to N-terminal luciferase (NLUC; Figure 6C). These findings indicated that GmPGL2 might affect RNA editing by interacting with GmMORF8, GmMORF9, and GmORRM6. However, the expression levels of GmMORF8, GmMORF9, and GmORRM6 were not significantly different between HD12 and *Gmpgl2* mutants during the two developmental stages (Figure 6D). It implied that the loss of E+ motif in *Gmpgl2* mutant might serve minor role among their interactions, which was not the binding domain of PPR protein with MORF and ORRM proteins (Hayes et al., 2015; Small et al., 2020).

## Discussion

As a post-transcriptional modification process, RNA editing fine-tunes gene expression and functions by altering specific nucleotides of a transcript (Oldenkott et al., 2020). In flowering plants, RNA editing generally changes cytidine to uridine in plastids and mitochondria, playing important roles in organelle biogenesis, adaptation to environmental changes, and signal transduction; PPR, MORF, and ORRM proteins play curial roles in plant RNA editing (Lu, 2018; Zhang et al., 2019; Small et al., 2020). Here, we found that the mutation of PLS-PPR protein, GmPGL2, caused the abnormal chloroplast development. GmPGL2 protein recognized nine RNA editing sites in six chloroplast transcripts in soybean, including *ndhB*-139627, -141281, -141424, -141650, *ndhD*-120618, *ndhE*-119873, *ndhF*-124681, *rps16*-56313, and *rps18*-66641. The editing capacity of GmPGL2 varies in these nine sites during the different stages of young leaf development. These six genes belong to the subunit of NDH complex and ribosomal protein. NdhB, NdhD, NdhE, and NdhF proteins are the subunit of complex of NDH, which is encoded by a combination of genes residing in the plastid and nuclear genomes. Rps16 and Rps18 proteins are the subunit of ribosomal proteins that translate the chloroplast-encoded proteins. The defects of post-transcriptional processing of *rps16* and *rps18* might decrease the chloroplast translation efficiency. GmPGL2 interacted with GmMORF8, GmMORF9, and GmORRM6 *in vitro* (Figure 6B). Therefore, we propose that GmPGL2 participates in soybean RNA editing together with GmMORFs and GmORRMs.

Previous studies in *Arabidopsis*, rice, and maize have demonstrated that a lack of RNA editing is often associated with changes in the expression levels of chloroplast genes (Jiang et al., 2018). In most cases, the expression level of NEP increased that of PEP decreased in mutants, such as *chloroplast biogenesis 19* (*clb19*), *pigment-deficient mutant1* (*pdm1*), and *pigment-defective mutant 2* (*pdm2*; Chateigner Boutin et al., 2008; Du et al., 2017). However, in the *Gmpgl2* mutant, the expression of NEP and PEP increased in developmental stages S1 and S2. Recently, this kind of changes has been observed in the knockout of *SLC1* gene, which encodes a P subgroup of PPR protein in rice (Lv et al., 2020). In *slc1* mutant, the transcript levels of 3 chloroplast ribosomal RNAs and 16 chloroplast development-related and photosynthesis-related genes were also significant increased. This phenomenon was attributed to preclude the intron splicing of *rps16* in the *slc1* mutant, which blocked the post-transcriptional processing and translation of *rps16*, and failed to assemble the normal 70S ribosomes (Lv et al., 2020). In our study, the *rps16*-56313 was the unique editing site, which was completely abolished in the *Gmpgl2* mutant. We infer that the lack of an edited functional Rps16 protein might compromise the function of the ribosome complex in chloroplasts. As a result, many of the chloroplast proteins may not be translated to the optimal levels, and this may be compensated in the *Gmpgl2* mutant by increased gene transcription.

In summary, the defective development of chloroplasts observed in the *Gmpgl2* mutant can be attributed to failure of RNA editing at the subunit of NDH complex and ribosomal protein-related genes, and *GmPGL2* plays a crucial role in chloroplast development and normal plant growth.

## Data Availability Statement

The original contributions presented in the study are publicly available. This data can be found at: NCBI repository, accession number: PRJNA660896 (https://www.ncbi.nlm.nih.gov/search/all/?term=PRJNA660896).

## Author Contributions

SY and QW conceived the project and designed the research. XF performed most of the experiments. YZ, HY, and JL helped to clone the gene. CZ carried out the REMSA experiment. KT and GL performed bioinformatics analysis. XF and SY prepared the figures and wrote the paper. All authors contributed to the article and approved the submitted version.

## Funding

The research was supported by the Chinese Academy of Sciences (ZDRW-ZS-2019-2 and XDA24030303) and the National Natural Science Foundation of China (31971901).

## Conflict of Interest

The authors declare that the research was conducted in the absence of any commercial or financial relationships that could be construed as a potential conflict of interest.

## Publisher’s Note

All claims expressed in this article are solely those of the authors and do not necessarily represent those of their affiliated organizations, or those of the publisher, the editors and the reviewers. Any product that may be evaluated in this article, or claim that may be made by its manufacturer, is not guaranteed or endorsed by the publisher.

## Footnotes

^1^ https://phytozome.jgi.doe.gov

^2^ http://www.cbs.dtu.dk/services/TargetP

^3^ http://yinlab.hzau.edu.cn/pprcode

^4^ https://www.ncbi.nlm.nih.gov/nuccore/NC_007942.1

^5^ http://www.cbs.dtu.dk/services/TargetP
