# Supplementary material for: GmPGL2, Encoding a Pentatricopeptide Repeat Protein, Is Essential for Chloroplast RNA Editing and Biogenesis in Soybean
Source: Front Plant Sci. 2021 Sep 9;12:690973. doi: 10.3389/fpls.2021.690973 (PMC8458969; doi:10.3389/fpls.2021.690973)
Supplement: Supplementary file 1 [file Data_Sheet_1.doc]

**SUPPLEMENTARY DATA**


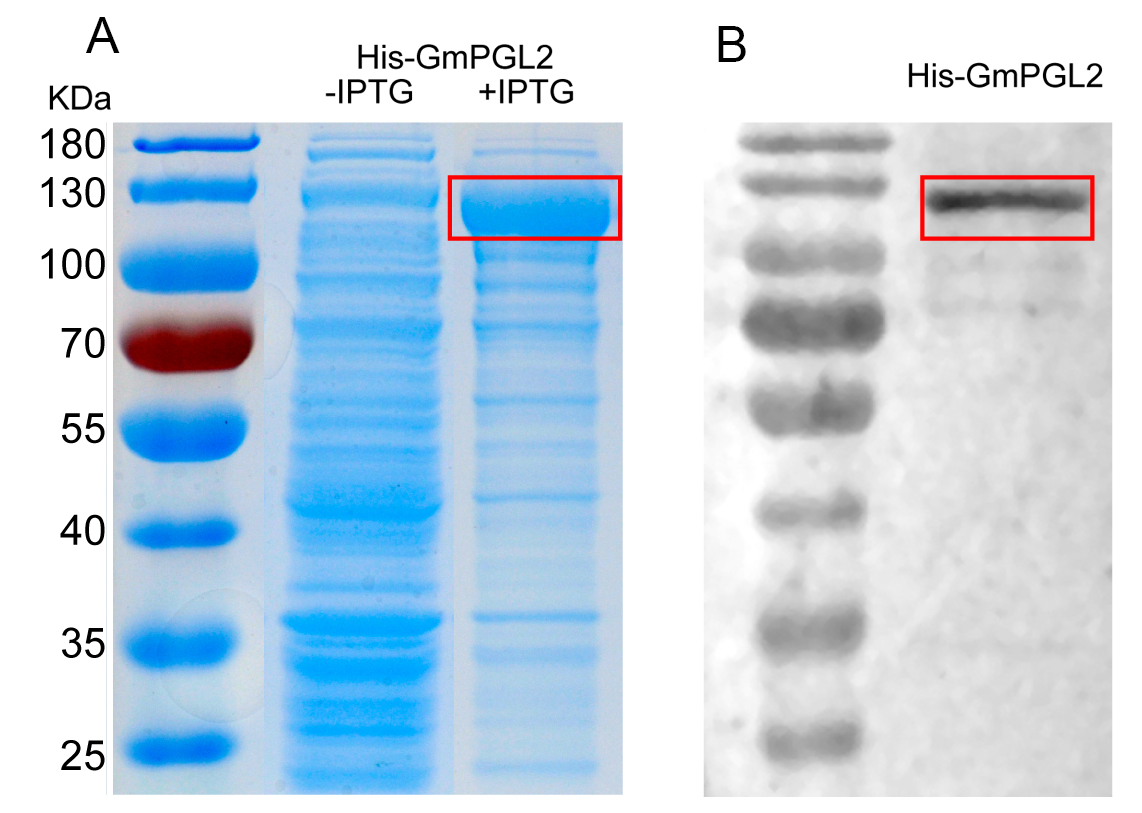


**Figure S1** The expressed and purified recombinant proteins of GmPGL2. **(A)** The expressed and purified recombinant proteins were validated by Coomassie brilliant blue staining. **(B)** Western blot of purified recombinant protein with His-antibody. The His-GmPGL2 recombinant proteins were indicated in red rectangular frames.

**
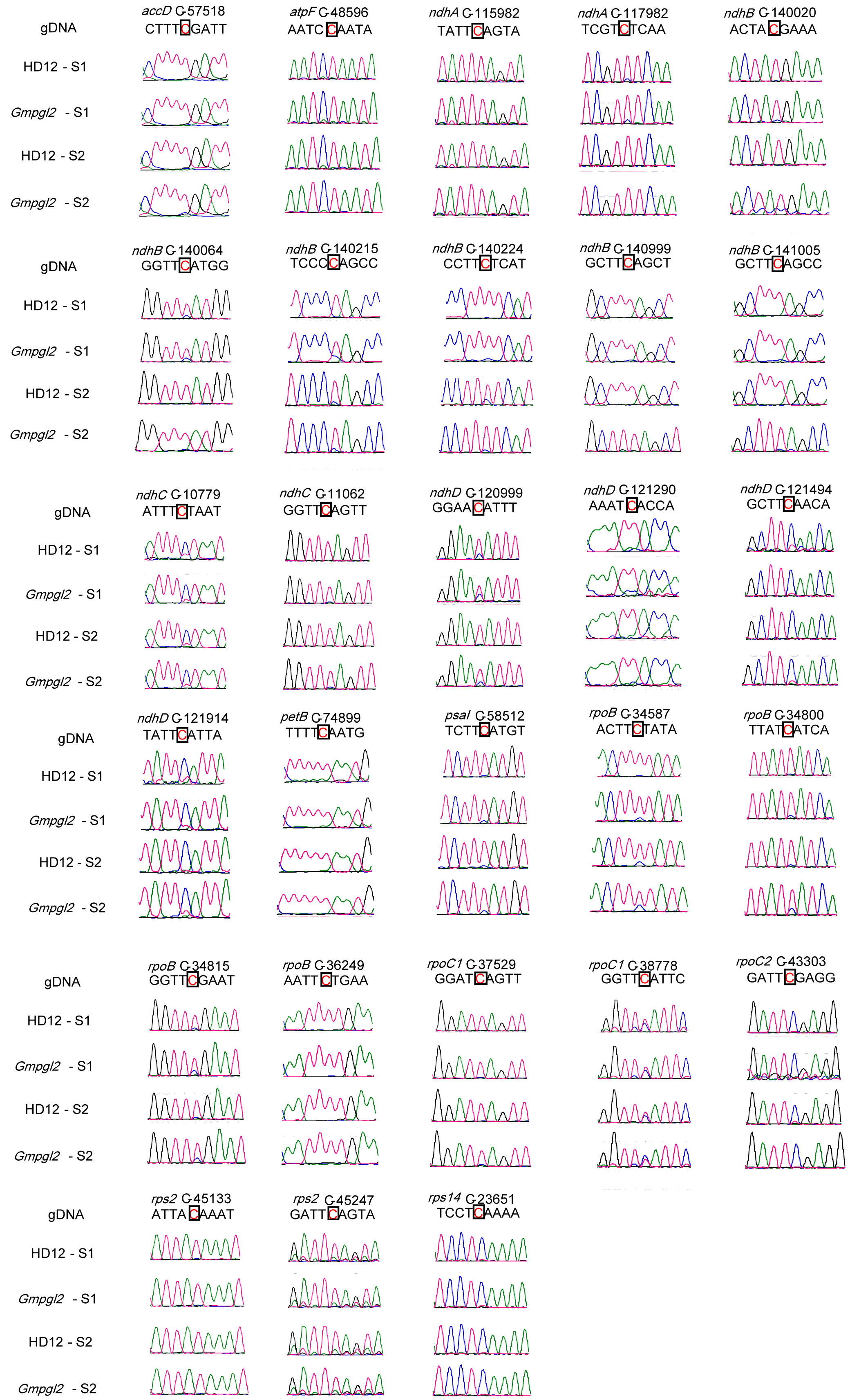
**

**Figure S2**  RNA editing sites confirmed by sequencing the RT-PCR products.

Table S1 Primers used in this study

| Primers | Forward and Revere primer sequence | Locus |
| --- | --- | --- |
| MOL 2345 | GCCTGGGCAATCTGTAAGCT GCCTCACAATCCGTATGTGGT | Chr05: 31703171 - 31703188 |
| MOL 2413 | CGATTTTCCGTCCTTGGTTTC CCGTGTTAAGATGAATTGGGGTT | Chr05: 31945477 - 31945496 |
| MOL 2417 | GGCCAAGAGTCAATTGCTTTAAC CGAATCTCACTTGGAATGCCT | Chr05: 31963364 - 31963384 |
| MOL 2419 | CGGCTTGTCAATCTCCTACATACT CGCGGATATGCTAGTTACCACTT | Chr05: 31988302 - 31988322 |
| MOL 2361 | CGCCTTGATGACCTCCTTATTG CGGTGTTGTCGTTGCTTTTGT | Chr05: 32099737 - 32099754 |
| MOL 2371 | CGGAAAGCATGGAAATGATGG CGGCCTTGTTCTTCTTGCACTC | Chr05: 32446539 - 32446556 |
| MOL 2409 | CGCGAAGGAAATGTCACTCAT CGCGTTGTATAGCAGCATCAGA | Chr05: 32618164 - 32618181 |
| MOL 2373 | CGGCCAATTACACGAATTAGAG GCGTTAGGGGCAAAAGAAAGT | Chr05: 32630772 - 32630789 |
| MOL 2441 | CTTGTGCAGTTTTGGCAATCA GGACTTCACGTTACCAGAGGGT | Chr05: 32149141 - 32149161 |
| MOL 2411 | CGCAATGTAATCCCCTGTCTC GCCGTGATCGATGTTATTGTTG | Chr05: 32592506 - 32592524 |
| OL 4284 | GCCGTTGGACCCTTCAATCTA GCCGTTCACAAATATGGAGCAG | Chr05: 32504462 - 32504445 |
| OL 4444 | CTGGAACCCACCCTATCAACA CCATATAACACGAGCCGACAA | Chr05: 32522301 - 32522282 |
| OL 4460 | CGCTTCATTTGTCTGCCCTCT CGCTTCCGATGCTTATGTTCTAT | Chr05: 32446885 - 32446902 |
| OL 4462 | GCGTTAAAATGGGGAACTGAG CCACACGAACACTAATGGAACTG | Chr05: 32464069 - 32464086 |
| OL 4464 | GCGCCTGTAAATTTTCTTTCTTAGA CGGCATTACGAAGATCATTACG | Chr05: 32456481 - 32456461 |
| OL 4466 | GGCGAGGAGCGTATTTATTGTC CGCATTCCCTCATACCCATCT | Chr05: 32480429 - 32480411 |
| OL 4468 | GGCGTGGCAAGTAGCAGATTAT CGGCTTCTAAGACATCTCCGAAT | Chr05: 32496213 - 32496195 |
| OL 4470 | CGCCTTCTTTTATACCGACCA GGCTGCATCTTAGATTGACCCA | Chr05: 32500168 - 32500151 |
| OL 4472 | CGAGAGTGACCGAAGGAGGG CCGAAGCCAATCAGAAACAGA | Chr05: 32515570 - 32515552 |
| OL 4474 | GCCGGTAATTGATGGGTAGTGT TCCGATCCTCCAAAGGAAGATA | Chr05: 32531608 - 32531590 |
| OL 4476 | GCGGTAAAACCCTACAAATCCAC CGGAAACCATTCTGTCCCATG | Chr05: 32556074 - 32556056 |
| OL 4478 | GCGAGAAAGCAAAGCAAAGG CCGTAGACGCCAGCATATCAA | Chr05: 32557418 - 32557435 |
| OL 4480 | CCGCGCTGGTTTAGATAAGAA GCTAACCGCTACCACCTTCC | Chr05: 32564108 - 32564125 |
| OL 4482 | TCGGTACCTGTGGAGCAATAGC GGCTGCGACGATAACAAATCTAAG | Chr05: 32568311 - 32568293 |
| OL 4484 | GCTTGCTTTTCCCGTTTCTC CGATGAATCGGCAGCTACAA | Chr05: 32578307 - 32578324 |
| OL5005 | AGGACCTCGAGAATTCATGAGGTTCTCCTTTGCAC CGTGGTTGTAAAGCTTCTAGATATTGGATGTTAACTCTTCT | Chr05: 32564138 - 32566367 |
| OL7594 | GGAATTCCATATGATGAGGTTCTCCTTTGCACAC CGAGCTCCTAGATATTGGATGTTAACTCTTCT | Chr05: 32564139 - 32566367 |
| OL7676 | GGAATTCCATATGATGAGGTTCTCCTTTGCAC CGAGCTCACCAACAACTGTATGCATAAAAAAACC | Chr05: 32564139 - 32564732 |
| OL 6199 | CGAACAGGCATAAATGTGGC CGATGAATCGGCAGCTACAA | *rpoA* |
| OL 6201 | TGAGGGACGGTCATAATAAGGT GAATAATCAACCCGTTTCCCAA | *rpoC1* |
| OL 6203 | CTCTGGGATTCCGTCAAGCT ACTTTGTTGTTCAGCATCTTGGAC | *rpoC2* |
| OL 6205 | AACGGGATTAACTGAGGCTGT TGCCCCCCATAAACTGAAA | *accD* |
| OL 6235 | CGAATCCCGCCTCAATCAT GCGACATGGACTAAATGCCCT | *psaB* |
| OL 6237 | GCTCGGTTAATAATATCAGCCCAG GCTTTAGGTATCAGCACTATGGC | *psbA* |
| OL 6239 | TCCCTCTGACCCTGTTCTTGAC CCACGAATTGGTTATTCCTAAACG | *psbB* |
| OL 6241 | TTGGGTACTTGATCGA CGCTAAGCCTACGTTACAGGC | *petD* |
| OL 6243 | GCAGCAGCTAATTCAGGACTCC TGAAGCATGTGTGCAGGCTC | *rbcL* |
| OL 6307 | CGCTCGTTGCGGGACTTA CCTCTTGAAAGAGAGGGGTGC | *rrn16* |
| OL 6309 | CACTTGGCTACCCAGCGTTTA GGTGTGGGCGTTAGAGCAT | *rrn23* |
| OL 6311 | GGTCTAATGAGAGGAATGGAAGTG GGACCTAAATTATCAATGGGCTC | *atpB* |
| OL 6313 | AACGATGGCTCTGATGGGC GAGTTTGCTGTGCTTCTTGTGG | *atpE* |
| OL 6315 | ACGAGATGTAACTCCTATGCCAC GCTGGTTTAGATCTATCCTAACCG | *rpoA* |
| OL6316 | GCTGGTTTAGATCTATCCTAACCG CAGACATACTCACTTATCACCCCA | *clpP* |
| OL 6331 | GTCATAGCAAATCCCGTTGCT TAGCACCAGGCACAACAGC | *petB* |
| OL 6333 | CAACTTTACCGCCCACTGCTA TTGGGTTGGGTTAAAAGCACG | *psaA* |
| OL 6335 | GTTGCTGGTGTATTGGGCG GAATTCCTTGTCGGCTCTCTGT | *psbD* |
| OL 6337 | AGGTTGGTTATTTGTCAGCACG TTGTGATCCTCGGGTCGTG | *psbE* |
| OL 6339 | CGTAATAATCTATCTTCGGGGGA GCTATGCGACTGGAAATTGATC | *rpoB* |
| OL 6341 | GTTCTCCGTTACTTGTGTGGATAAG GAGTATTGCTCCCATTGGACTTC | *ycf3* |
| OL 6343 | GCTGCTCGTAGACCACCCA CCACTCCAGTCGTTGCTTTTCT | *ndhA* |
| OL 6375 | CAGAAGAAGATGCCATTCGTTTG CCCGATTAGCAAGGGACCA | *ndhB* |
| OL 6377 | CGAAATTGTAACCAAGCATCACC TGACTTTTACTGGAGCTTCGGA | *ndhC* |
| OL 6379 | ATTTGGATTCTCTAGTAACCGGC CGAGCATGCACTTGTTTTGAG | *ndhD* |
| OL 6381 | TCAAGACACATAAGGGCTCTAACC GCTCAGTCGCTAAATCCGC | *ndhE* |
| OL 6383 | GTAGCAGCATGTATAAGGGCG GGACTGTTGGGGATGGAATTAC | *ndhF* |
| OL 7415 | CTTCACATTTTGAATTGCACCG CAGCAATCTTTGTCGTTTTCCC | *rps15* |
| OL7217 | TTCCTTGAACGAGGCGCT CAAGTCATCCACACCCCCT | *rps16* |
| OL7219 | GGAACTGTTCAGGACA CATAAAAACACCAGCC | *rps16* |
| OL7221 | TCTTGGATAATGAGTCGGACC GACAAAATTATCAAAGATCGGC | *rpoC2* |
| OL7225 | GCCTTGAGAAAGGGCAGATG CCCTGTCGAGGTAAGGAACC | *ycf2* |
| OL7234 | CCCCAGGAATTTTAATTGC AAGCAACGACTGGAGTGG | *ndhB* |
| OL7238 | GTTTGGGTACTTGATCGATTCAC CTCAAATATCCTTGATCATGAGACA | *ndhF* |
| OL7260 | CAAGCTCTAGCAGACGGAACA GGGAGCAATACTCGAAATAGCA | *ndhA* |
| OL7268 | CGGTTTTTCTTTATCCTTGGGC CTAATGCCCCCTTTCGCC | *ndhC* |
| OL7272 | GAGCGCGGGTTTTTCTAGTC ACCCCCGACTATTGGCAA | *ndhD* |
| OL7278 | ACATTCCTTTAATGAATTCTCTGGG GGGCTTTGCAATATTTGATTGA | *rpoB* |
| OL7280 | CATCAACAACTCCGAATTGGAT CCAAGCACTTATTTGTTGAGGAGA | *rpoC1* |
| OL7284 | TATCATTTGATTCGCCGATCC CACTTAACGACGGAACTTTGCT | *rps14* |
| OL7286 | GGATTTGGTAGTTTGATCGTGGA CAAGACGTTCTTCGAACCAATC | *petB* |
| OL7288 | GATGTACGGTCTAATGAGGCTACT GAACCATATAGCCAAGAGAAACC | *ndhB* |
| OL7291 | CAGCTGCTGAAGCAGCTATCG ACGATAAATTGAGGAAACAATAGCC | *ndhE* |
| OL7299 | ACAATTGCTATAAAACAAGCTCGT GTTGACTCGCTTTTTTCAAATTG | *rps18* |
| OL7301 | GGATCCACACAAGGAAAAGCAC GGATGCCCCCAATAACCAAC | *rps7* |
| OL7303 | GCTCATTCTATATTTTCTCCCTGG AATTGCGTTGACCAAGAGATGT | *ndhD* |
| OL7350 | CGGCAATTGCAATGGCTT TGGGGAATAAAAAACCCCAGA | *psaI* |
| OL7352 | CAGCAAGTGAGGGAAAACAATTC CTCTTATTGCAGCCCGTGCTA | *rps2* |
| OL7356 | CAAAAGTATTCGGTTATTGGGGA CCAATGCTTGATTTCTGTCCTAG | *rpl23* |
| OL7360 | TGTGGGACAATCTACCTTAACTCG GACCAGAAATGCCTTGCTTACG | *petB* |
| OL7362 | GGGCAGTTCAGATAGAATCGAAC GATTCCAAGTACCCGAATCAATC | *accD* |
| OL7364 | CCGGAAGTTTCGGGTTTAATAC CACTCCCTTTCCAAAAAAAACC | *atpF* |
| OL10567  OL11254  OL11255  OL11256  OL11257  OL11258  OL11259  OL11260  OL11261  OL13194  OL13195  rps16-FAM  ndhB-141281-FAM  ndhB-141424-FAM  rps14-FAM | ATGGCCATGGAGGCCGAATTCATGAGGTTCTCCTTTGCACACTG ACGGGGGACGAGCTCGGTACCATGAGGTTCTCCTTTGCAC  AACATCGTATGGGTAGTCGACGATATTGGATGTTAACTCTTC  ACGGGGGACGAGCTCGGTACCATGGCGACTCAGATCCTC  CGCGTACGAGATCTGGTCGACCCCAGAATATGGGTTCC  ACGGGGGACGAGCTCGGTACCATGGCGACGGTGACG  CGCGTACGAGATCTGGTCGACTGTAGAGGATGAATCCGA  ACGGGGGACGAGCTCGGTACCATGGCTAACAACGTCG  CGCGTACGAGATCTGGTCGACCAATCTAGTGTCTTCCTG  GGTGGTATCGAAGGTAGGCATATGATGACTCAACCACTCATCGA  CTGCAGGTCGACAAGCTTGAATTCGATATTGGATGTTAACTCTTC  CCUACAGGAACUGUUCAGGACAUUUCAAAAAGGGC-FAM  CUAGGAACUUUUGCUUGCAUUGUAUCAUUUGGUCU-FAM  ACUAUCAGGUUUUUUCGGAAAACUUCAUUUAUUCU-FAM  AAAUAUCAUUUGAUUCGCCGAUCCUCAAAAAAAGA-FAM |  |

Table S2 Candidate genes in the *Gmpgl2* mutant

| Genes | Function |
| --- | --- |
| *Glyma.05G131500* | chromatin remodeling 4 |
| *Glyma.05G131600* | unknown |
| *Glyma.05G131700* | unknown |
| *Glyma.05G131800* | K+ uptake permease 11 |
| *Glyma.05G131900* | Tetratricopeptide repeat (TPR)-like superfamily protein |
| *Glyma.05G132000* | FtsH extracellular protease family |
| *Glyma.05G132100* | N-acetylglucosamine-1-phosphate uridylyltransferase 1 |
| *Glyma.05G132200* | Inositol monophosphatase family protein |
| *Glyma.05G132300* | PLATZ transcription factor family protein |
| *Glyma.05G132400* | unknown |
| *Glyma.05G132500* | Ribosomal protein L35Ae family protein |
| *Glyma.05G132600* | thioredoxin O1 |
| *Glyma.05G132700* | Tetratricopeptide repeat (TPR)-like superfamily protein |
| *Glyma.05G132800* | Ribosomal L27e protein family |
| *Glyma.05G132900* | copper-exporting ATPase |
